# BattleScars: Global Conflicts and Environmental Health

**DOI:** 10.1289/ehp.112-a994

**Published:** 2004-12

**Authors:** Valerie J. Brown

War is as old as humanity, but the study of its environmental health effects is just beginning. Age-old problems still follow war—lack of food, shelter, water, and sanitation, risk of infectious diseases, and psychological trauma. But war today, in all its modern permutations, can also saddle populations with new threats from industrial and military chemicals, pesticides, and radiation.

Modern conflicts show a fundamental departure from the form of earlier wars. The Nobel Foundation report *Wars in the 20th Century and Nobel Peace Prize Statistics* states, “From 1900 to 1910, wars of all categories were represented rather evenly, whereas from 1990 to 2000 most were civil wars.” Between 1945 and 1975 many former European colonies waged wars of independence in Latin America, Africa, and Asia. “Today there are few interstate wars with clearly defined parties, but civil wars have become increasingly internationalised,” states the report. “Few internal wars today take place without the intervention of foreign states.”

The post–Cold War world is split by development inequities, competition for control of natural resources, and seemingly intractable ethnic and religious divisions. Today, more than ever, conflict is a tangled interplay of social, political, and economic factors. In a speech delivered to the United Nations on 5 October 2004 titled “Development and Conflict,” Paul Collier of the Center for the Study of African Economies at Oxford University noted that the more dependent a country is on the export of natural resources, the more vulnerable it is to civil war, and that doubling the per capita income halves the risk of conflict. [For more on the connection between conflict and natural resources, see “Global Resources: Abuse, Scarcity, and Insecurity,” *EHP* 112:A168–A175 (2004)].

Wars are costly, too. Civil war in a poor country lasts an average of 10 years and costs $50 billion. More than half this cost is borne by neighboring countries, which often see influxes of fleeing refugees and combatants, Collier said.

Perhaps the most important change in warfare, from the perspective of the environment, is the fact that wars are no longer limited to a designated field and clearly identifiable combatants. Instead, they may rage in urban streets and village squares, on cultivated land, or along highways, and the fighters may emerge from and blend into the civilian population. Because conflicts are no longer cordoned off in specified combat zones, but are now played out in everyday human environments, the environmental health consequences of war increase exponentially.

## The Effects of Destabilization

The invasion of modern warfare into urban areas means millions of people can be rapidly displaced. Some of these people become refugees in other countries, but many others stay in-country as so-called internally displaced persons (IDPs). Globally, the movement of refugees and IDPs is a fluid, indeed tidal phenomenon. The country of origin for the largest number of refugees is Afghanistan, with about 2.1 million people having fled by the end of 2003, according to the UN High Commissioner for Refugees report *2003 Global Refugee Trends*. Most Afghan refugees go to neighboring Pakistan (which hosts about 12% of all refugees under the protection of the UN High Commissioner for Refugees) and Iran.

Despite the seemingly constant number of conflicts around the world and the many populations of refugees and IDPs, the *2003 Global Refugee Trends* report noted a drop of just over 3 million in such populations from 2002 to 2003. An “almost unprecedented level of voluntary repatriation” was observed in 2002 and 2003, with 3.5 million refugees going home. The number of IDPs has also decreased in some regions, including Bosnia and Herzegovina, Angola, The Former Yugoslav Republic of Macedonia, and the Democratic Republic of the Congo.

But the report also noted significant increases in refugees moving from Sudan to Chad and from Liberia to Cote d’Ivoire, Guinea, Sierra Leone, and Ghana. And a total of at least 1 million IDPs remain in Azerbaijan, Georgia, the Russian Federation, and Serbia and Montenegro. Colombia and Liberia each saw more than 250,000 more IDPs in 2003.

By far the greatest danger to the greatest number of people in conflict areas and those fleeing violence is the lack of life’s most basic necessities: potable water, food, shelter, and sanitation facilities. Crowded quarters make infectious disease outbreaks inevitable. Stressed by trauma and malnutrition, and without adequate medical care, refugees cannot fight off cholera, typhus, hepatitis, scabies, and numerous other contagious ailments.

Carol Smedberg, an emergency medical technician who volunteers with the Portland, Oregon–based Northwest Medical Teams, visited Liberian IDP camps in September 2004. “The main problem is the water,” Smedberg says. “Normally we tell people to drink more water, but there the water is the cause [of most of the health problems].” People have only charcoal briquettes for fuel, Smedberg says, and it is almost impossible to boil their drinking water, which is taken out of a stream that is also used as a toilet. Chicken pox breaks out about every two weeks as new people arrive in the camps, according to Smedberg.

Relatively developed countries are at just as much risk of war-related environmental health problems as the developing world. According to the *GEO Year Book 2003* published by the UN Environment Programme (UNEP), unreliable electricity supplies in Iraq have caused sewage treatment equipment to stop working, sending raw sewage and industrial waste directly into the Tigris River, Baghdad’s only source of water, as well as other bodies of water. On 25 September 2004 *The New York Times* reported that water and sewerage failures had contributed to an outbreak of at least 200 hepatitis E cases and 5 deaths. Like other forms of the disease, hepatitis E causes fever, jaundice, fatigue, nausea, and vomiting; it is especially threatening to pregnant women and fetuses.

Iraq’s problems don’t end there. In a *Lancet* paper published online on 29 October 2004, Les Roberts of the Johns Hopkins Center for International Emergency Disaster and Refugee Studies reported that the risk of death had more than doubled after the 2003 U.S. invasion. The major post-invasion cause of death was violence, which was widespread and attributed mainly to coalition air strikes. Excess deaths were estimated to be at least 100,000, with most victims being women and children. And a national assessment conducted by the new Iraqi government’s health ministry reported 5,460 cases of typhoid in the first three months of 2004, according to a 13 October 2004 article published in *Nature* online. The Iraqi report also said mumps, measles, and other infectious diseases were ravaging the country’s children, one-third of whom are chronically malnourished. In fact, the report said, Iraq’s once relatively robust overall state of health is now comparable to that of Yemen and Afghanistan, where citizens face very high infant mortality and little access to clean water and sanitation services.

It can be difficult, sometimes impossible, to deliver aid to conflict-ridden regions. In an April 2004 country brief on Sudan, officials with the UN World Food Program (WFP) estimated that food assistance was necessary for 1.18 million Sudanese who were chronically malnourished due to drought, floods, and war. Aid was begun but suspended in mid-October after two Save the Children aid workers were killed by a landmine, and the WFP decided the security situation was too unstable to put its aid workers at further risk.

Many of these same problems exist in the Chechnya conflict. By 2003 about 260,000 Chechens had set up camps in the adjacent Republic of Ingushetia in farm fields and factory grounds, living in leaky tents with inadequate protection from the cold. Tuberculosis was common.

The New York City–based International Rescue Committee (IRC) has set up or repaired 66 potable water supply points, collected garbage, and serviced latrines in Ingushetia, but Ingush authorities have restricted the amount and type of aid humanitarian groups could provide to refugees in Ingushetia. For example, according to a June 2003 press release by the international medical aid agency Médecins Sans Frontières (MSF), Ingush authorities had just that month suddenly declared an MSF temporary shelter project illegal and barred Chechen refugees from moving in.

Despite this pressure, most Chechen refugees are loath to return to Chechnya, where conditions are very dangerous, housing is almost nonexistent, and services have broken down. To help ease the situation in Chechnya, the IRC has for the last three years been trucking water to 20,000 Chechens in Grozny. And as of 2003 the organization had built 35 water reservoirs to be hooked up to the city’s water mains. The IRC also builds and maintains latrines in Grozny, conducts pest control activities, and resurrects homes, including making repairs to electrical and gas lines.

## Weapons of War I: Landmines

Landmines have been in widespread military use since World War II, and the UN estimates there are 60–80 million laid around the world, many in places where conflict has long since ceased. Such landmines can destroy lives and societies for generations. According to the *Landmine Monitor Report 2003*, a publication of the International Campaign to Ban Landmines (ICBL), there are an estimated 200–215 million landmines currently stockpiled by 78 countries. All but about 10 million of those landmines are in nations that are not parties to the Mine Ban Treaty, an international convention that requires signatories to destroy their stockpiles within 4 years and clear all laid landmines within 10 years. Among these nonsignatories are China (home to an estimated 110 million landmines), Russia (with 50 million), the United States (with 10.4 million), and Pakistan (with 6 million). The *Landmine Monitor Report 2003* also states that mines cause 15,000–20,000 new deaths and injuries per year (most victims are male civilians). Landmine conditions are dire and worsening in several countries, such as Chechnya and Nepal.

In more than 80 countries landmines make land unusable and impede the post-conflict return to functioning economies and social life. Children who have lost limbs generally need a new prosthesis every year to keep up with their growth. Survivors can have great difficulty working, particularly in rural and agricultural communities. And strained medical systems are easily overwhelmed by victims’ need for continuing care.

In Thailand, an area of about 2,557 square miles is contaminated with landmines, affecting half a million people, according to a Kingdom of Thailand Landmine Impact Survey completed in 2001. The densest concentration of landmines lies along the border with Cambodia. Most are distributed in hilly forest areas, preventing traditional uses of the forest, such as food- and wood-gathering, and making decommissioning very difficult.

But ICBL coordinator Liz Bernstein says the general trend is toward a lessening of the scale of devastation, thanks to the Mine Ban Treaty and other ban movements. The treaty has been ratified by 143 countries. More than half the countries where landmines are deployed are at peace, enabling decommissioning to begin. Bernstein says, “When we began [working on the treaty] there were 54 countries producing landmines; today there are about a dozen. Now there’s virtually no trade in landmines. The only governments we found last year actively using them on a daily basis were Russia in Chechnya and Burma/Myanmar, where there is a civil war.”

## Weapons of War II: Depleted Uranium

Probably the most inflammatory war-related environmental health issue is that of depleted uranium (DU), which is the remnant of uranium left after U-235 (the isotope used in nuclear power generation and bomb production) is largely removed. Because of its high density, DU is used both in armor-piercing shells and in tank armor itself. DU ignites upon impact, sending a fine black powder of mixed soluble and insoluble uranium oxides into the air. The North Atlantic Treaty Organisation (NATO) and the U.S. military fired DU weapons during the 1991 Gulf War and against the Serbs in the Balkan crises of 1994–1995 and 1999. The United States also used DU in the 2003 Iraq war, and the British used small amounts in the Iraq and Kuwait wars in 1991 and in 2003.

Uranium is everywhere in the environment, but generally at low concentrations. Most human exposure is through ingestion via food and water. DU is about 60% as radioactive as naturally occurring uranium, and is chemically toxic as well. If ingested, DU behaves very similarly to ambient natural uranium, which the body clears fairly rapidly through urine and feces. However, the insoluble oxides of DU can become lodged in the body by inhalation or as shrapnel fragments. The radioactivity and chemotoxicity of DU may cause serious health effects in these circumstances. Large doses by any route of exposure can cause kidney and gene damage.

It is not clear how many people were exposed in the Balkans or in Iraq, or how much DU they were exposed to. Dan Fahey, a Ph.D. candidate at the University of California, Berkeley, and a DU policy analyst, says, “We don’t have good data. The Pentagon once said thousands of people [in the Gulf War] might have been unnecessarily exposed, and then backtracked to about nine hundred people.” According to an Army spokeswoman who spoke on condition of anonymity, no estimate of DU exposures in the 2003 Iraq war is available, but DU was used only during the invasion phase when the Iraqis were using tanks. Therefore, the U.S. Army believes exposures to be few in number and low-level.

Since the 1999 Kosovo war, allegations have flown that DU causes cancers such as Hodgkin lymphoma as well as immune, neurological, and reproductive damage. There is not a large body of research on these links. But a number of published *in vitro* and rodent studies by Alexandra Miller and colleagues at the Armed Forces Radiobiology Research Institute in Bethesda, Maryland, (including one published in the August 1998 issue of *EHP*) suggest that DU can change human cells to a tumor-inducing phenotype and cause oxidative DNA damage. In rodents DU was shown to migrate from the implant site to bone, kidney, muscle, and liver tissue; to alter the hippocampus; to cross the placental barrier; and to enter fetal tissue. Although DU is a weak alpha emitter, the bystander effect—in which untargeted cells surrounding an irradiated cell show damage similar to that of the target cell—may also be part of DU’s effects.

Nevertheless, the Army maintains that veterans with embedded DU shrapnel are not at risk for adverse effects. The Army spokeswoman says the government is tracking 70 Gulf War veterans who still carry DU shrapnel. “They have no ill effects from the shrapnel that came from DU rounds,” she says. “Depleted uranium has been studied probably more than any other substance used in warfare and has not been demonstrated to have ill effects. There have been thirty-five children born to these veterans, and none has a birth defect.”

Because of the dearth of good epidemiological DU studies, Fahey says the government’s highest priority should be to track a large number of DU-exposed Gulf War veterans. “If the latency period for DU is ten to thirty years,” Fahey says, “now is the time to be monitoring these nine hundred people.”

## Weapons of War III: Herbicides

Herbicides as a weapon first came on to the radar during the Vietnam War, when some 19 million gallons of chemicals were sprayed on Vietnam and Laos to strip away enemy cover and destroy crops. The different herbicide formulations, known collectively today as Agent Orange, were contaminated with 2,3,7,8-tetrachlorodibenzo-*p*-dioxin, a known human carcinogen. Decades after spraying ended, a quarter of this persistent toxicant remains in the Vietnamese environment, and the NIEHS and the Vietnamese government are working together to fully characterize the health effects of exposure to Agent Orange.

Today, herbicides play a major role in the Colombian drug war, another example of the changed nature of modern war. Several insurgent groups have been battling the Colombian government in a protracted and bloody civil war. The war has provided narcotics growers and processors uncontrolled zones in which they can flourish; insurgents and narcotics cartels have formed alliances. According to the U.S. embassy in Bogotá, most of the cocaine and heroin on the U.S. market comes from Colombia. To stop this flood, the U.S. and Colombian governments have jointly developed and implemented the Plan Colombia eradication program.

A major component of the plan is aerial spraying of herbicide on coca and poppy plants, which began in 2000. The main ingredient is glyphosate, widely used as a weed killer in several formulations of Monsanto’s Roundup and in other products, and the most commonly used commercial herbicide in the world. According to the National Pesticide Telecommunications Network, glyphosate causes mild eye and skin irritation and digestive and respiratory irritation when ingested, and has not been shown to cause reproductive damage or cancer in humans or wildlife.

However, many Colombian farmers in sprayed areas report significant skin problems, headaches, vomiting, miscarriages, and deaths of small children—effects that they attribute to the spraying. Residents of the sprayed areas are not told when spraying will occur for security reasons, so they cannot take any steps to protect themselves, their families, their crops, or their livestock.

The Colombian government and the U.S. embassy have a monitoring program in place to investigate all complaints related to spraying, from reports of planes spraying legitimate crops to glyphosate causing health problems. Half of the nearly 5,000 complaints received to date have been rejected as invalid, because it was determined that spraying did not take place in the areas in question on the dates claimed. Another 1,680 cases are under review by the government/embassy team. Compensation for lost crops has been paid in 12 cases and, according to press officer Paul Watzlavick of the U.S. embassy, there have been no cases where it was determined that spraying caused adverse health effects in humans or animals.

There is some controversy over research being done on effects of the spraying. In 2001 Colombian toxicologist Camilo Uribe led an embassy–funded study of the spray program’s health effects in the town of Aponte, which concluded that the observed health problems in the village—mainly skin problems and eye inflammation—were not related to the spray program. In a critique of the study, Rachel Massey, a fellow of the Institute for Science and Interdisciplinary Studies in Amherst, Massachusetts, noted that the study did not follow normal epidemiological protocols, such as indicating the total number of patient records from which the samples were drawn and how cases were selected. Moreover, the Uribe report itself noted that 7 of 10 nearby municipalities reported increases in patients seeking help for symptoms that their community doctors thought might be related to the spraying. One of these towns, San Pablo, had 50 cases of dermatitis, conjunctivitis, respiratory conditions, and digestive problems after nearby spraying.

The U.S. government says the narcotics cartels are responsible for more environmental degradation and toxic chemical exposures than the spraying program is. Says Watzlavick, “The coca growers use tons of pesticides and herbicides on their fields in addition to tons of other chemicals to produce cocaine. These are the chemicals that we see ending up in the water systems.” Chemicals used in drug processing include kerosene, sulfuric acid, ammonia, acetone, and others, along with the herbicides paraquat and 2,4-D. Chemicals and waste products are often dumped in water or left on the ground. Activists don’t deny the likely drug-related exposures, but believe Colombians are suffering additive effects from both kinds of exposures.

## Industrial Pollution: During Conflict and After

In the first Gulf War in 1991, Iraqi soldiers set more than 600 Kuwaiti oil wells afire. Vast columns of black smoke billowed into the sky for weeks. In an apparent attempt to deter invading forces during this war, Iraq built a 47-inch pipeline into western Kuwait and criss-crossed the area with trenches into which oil was pumped and set afire, according to the Center for Research and Studies on Kuwait, a Kuwaiti nongovernmental organization. Sabotage of Iraq’s own oil production facilities and pipelines began with the onset of war in 2003. Potentially toxic components of oil fires include polycyclic aromatic hydrocarbons (PAHs), metals, sulfur dioxide, ozone, and lead. Health effects from inhaling these components include cancer (from PAHs), asthma and airway inflammation (from ozone), burning of respiratory tissues and airway obstruction (from sulfur dioxide), and high blood pressure and kidney damage (from lead).

A 2000 Department of Defense study of Gulf War soldiers’ exposure to oil fires concluded that “except for particulate matter, air contaminants were below levels established [by the U.S. Environmental Protection Agency] to protect the health of the general population” and that no long-term damage was done, although some veterans blamed the oil fires for worsening their existing asthma and bronchitis, as well as for skin rashes and shortness of breath. According to the report, the Iraq–Kuwait region normally has some of the world’s highest levels of suspended particulate matter in the air, partly from the sandstorms common there; 18% of Kuwaiti civilians have respiratory problems, about three times the rate in the United States. Some of the soldiers’ symptoms might therefore have resulted from the combination of chemical and particulate exposures.

Urban and industrial areas present other serious environmental health risks in wartime. During the 1999 Kosovo war, NATO and U.S. planes repeatedly bombed several sites in Serbia, including the industrial complex at Panc evo, a town of 80,000 located a few miles northeast of Belgrade. The Panc evo complex includes a fertilizer plant, a petrochemical factory, and an oil refinery; wastewater from all the facilities drains into the Danube River through a canal. The joint UNEP/UN Center for Human Settlements Balkan Task Force issued a postwar environmental assessment concluding that although the war had triggered major chemical releases, the industrial sites were already seriously polluted.

The assessment team estimated that about 2,314 tons of the solvent ethylene dichloride and more than 88 tons of metallic mercury leaked out of the petrochemical plant during the war. Ethylene dichloride is a known human carcinogen, according to the National Toxicology Program, while mercury causes neurological and developmental damage. U.S. bombs burned about 500 tons of vinyl chloride monomer—also listed as a carcinogen by the National Toxicology Program—releasing dioxins, carbon monoxide, and polycyclic aromatic hydrocarbons. Fearful of further explosions, the fertilizer plant managers released about 275 tons of liquid ammonia into the canal. Though not identified as a carcinogen, ammonia can cause severe tissue burns and even blindness when inhaled or ingested, according to the Agency for Toxic Substances and Disease Registry.

Little information is available on disease patterns near the complex, but locals called a common ailment of site workers “Panc evo cancer.” Task force analysts think the condition was actually angiosarcoma of the liver resulting from high vinyl chloride monomer exposure.

## The Wages of War

As the character of modern war has changed—becoming less of a “formal” battle between clearly designated opponents in a specified area and turning more to intermittent yet long-term conflicts among insurgents, militias, and government forces—civilians get caught in the cross-fire more frequently. They turn into refugees and IDPs, vulnerable not only to physical violence, malnutrition, and disease, but to chemical and radioactive exposures as well. Their living environments may remain contaminated with industrial and military chemicals and munitions emitting radionuclides long after conflicts have ceased.

Few military groups track civilian casualties, and those who do generally underestimate them. For example, although the United States does not have an official estimate of civilian casualties, research suggests that the U.S. action in Iraq has led directly to the deaths of an estimated 100,000 Iraqis, mostly women and children. Humanitarian aid systems designed to help people after natural disasters are not able to function properly in combat environments. Thus, in severely war-torn regions, help is often only sporadic as conditions permit, or is simply not available.

There are some encouraging signs of progress to be found in the record of the world’s wars. One is the fact that landmines are falling into disuse. The Mine Ban Treaty came about largely because landmine activists, frustrated at the slow pace of UN negotiations, held their own summit in Canada, drafted a convention, and began collecting signatures. The UN has now adopted the convention, and more countries continue to ratify the treaty. Some 31 million stockpiled mines have been destroyed since the campaign began, and the number of countries producing landmines has dropped from 54 to 12. Perhaps the landmine campaign may serve as a model for mitigating other types of war damage and trauma.

## Figures and Tables

**Figure:**
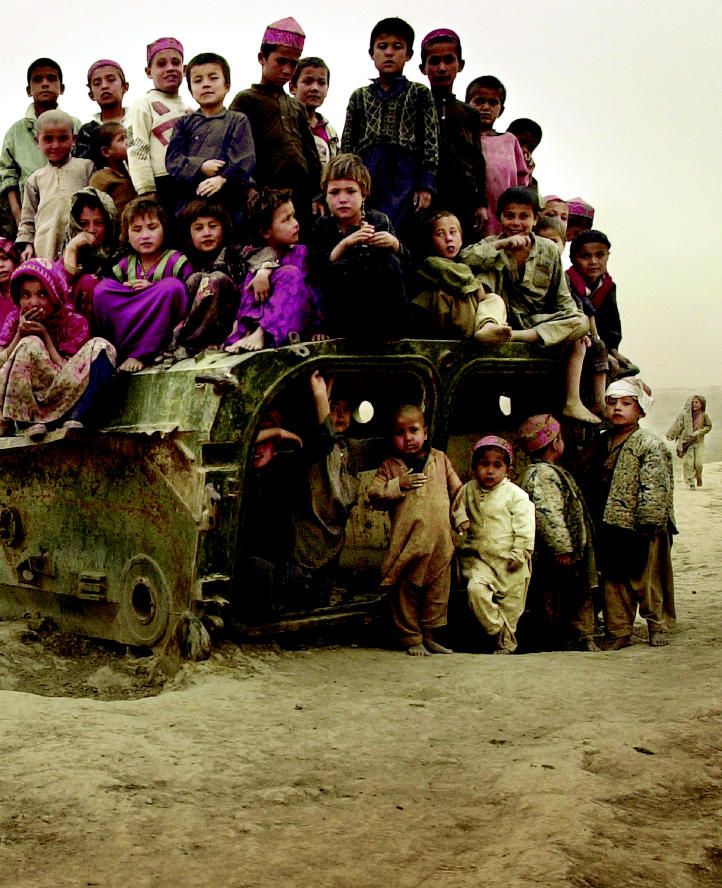
**A brutal reality.** Children in the Nawabad refugee camp in Afghanistan sit on a piece of abandoned military hardware. All across Afghanistan the detritus of war has become a plaything for generations of children.

**Figure:**
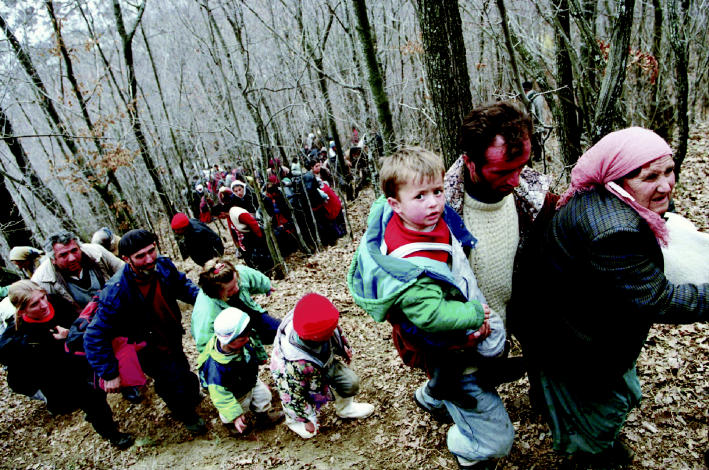
**Not yet out of the woods.** Ethnic Albanian families leave the woods below Gajre to head to a safer location. They hid in the woods for three days while Serbian forces shelled their villages.

**Figure:**
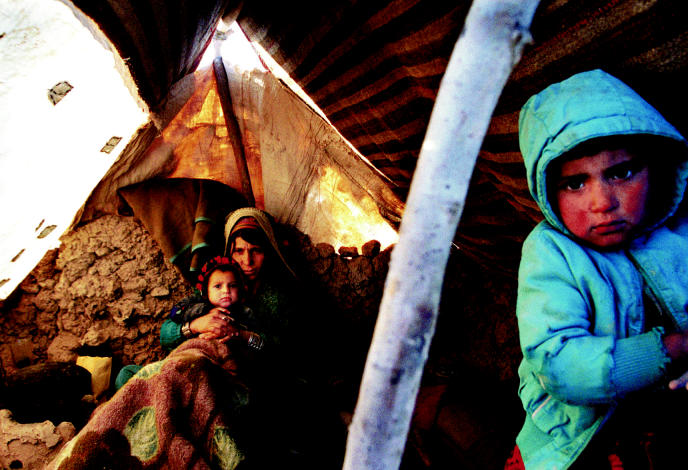
**True environmental exposure.** A refugee family in Sar-e-Pol, Afghanistan, huddles together against the cold in a makeshift shelter. Médecins Sans Frontières estimates there are 3,500 families living in tents made of nothing but cloth and plastic, in dire need of water and sanitation.

**Figure:**
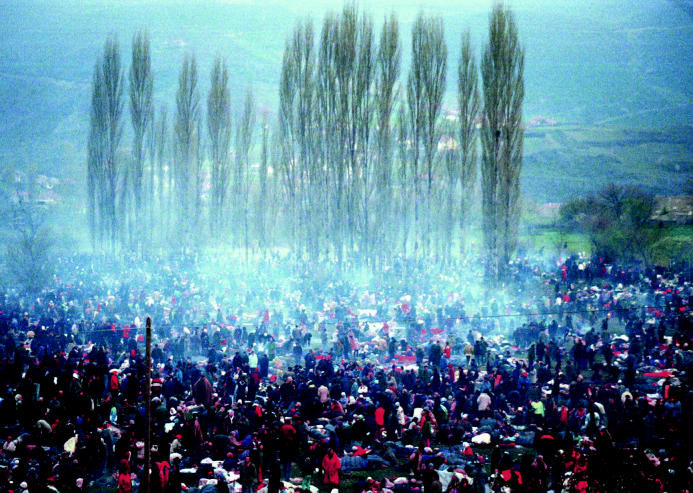
**Sea of refugees.** During the 1999 war, ethnic Albanian inhabitants of Pris tina waited in a field near the Macedonian border at Blace after being forced from their city by Serbian forces.

**Figure:**
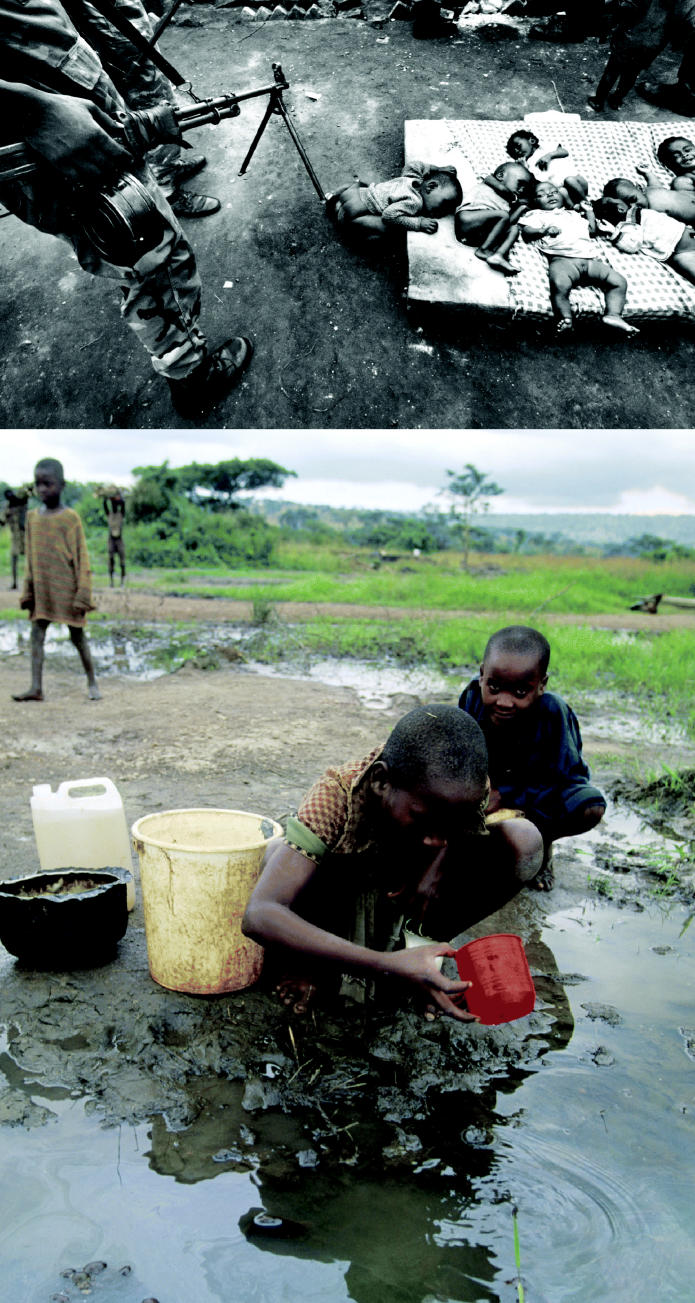
**Babes and arms.** (top) At Kibeho camp in Ngara, Tanzania, soldiers keep watch over some of the 1,000 children orphaned in the 1994 massacre of 4,000 Hutus by the Tutsi army of Rwanda. (bottom) Refugee children at the same camp must fetch drinking water from a muddy pond contaminated with fuel.

**Figure:**
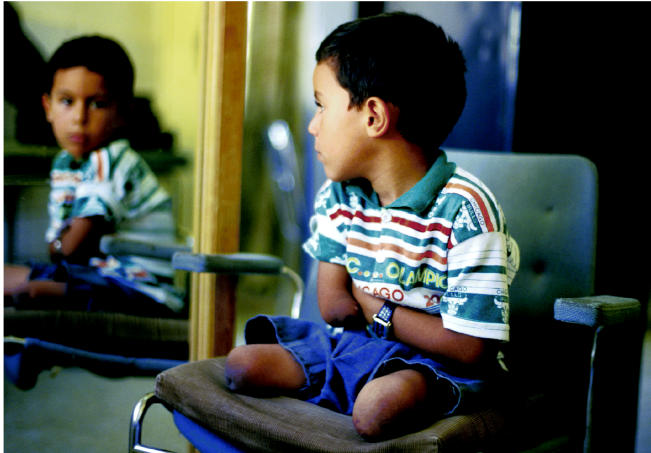

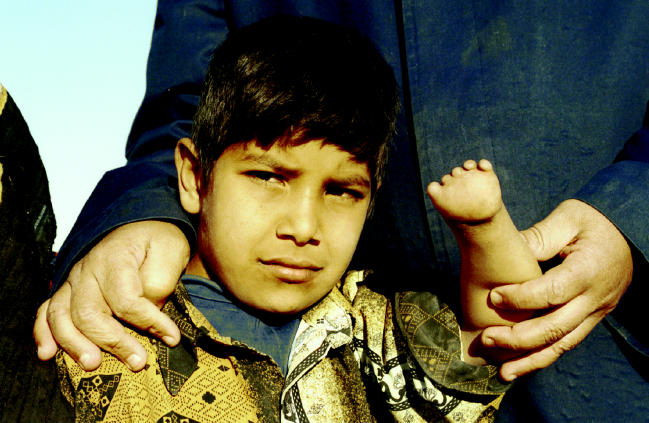
**Innocent victims.** (top) A child landmine victim in Kurdistan waits to be fitted with prostheses at a center for disabled children. (bottom) Congenital birth defects among Iraqi children are believed to be connected to the use of depleted uranium munitions by Allied forces during the first Gulf War.

**Figure:**
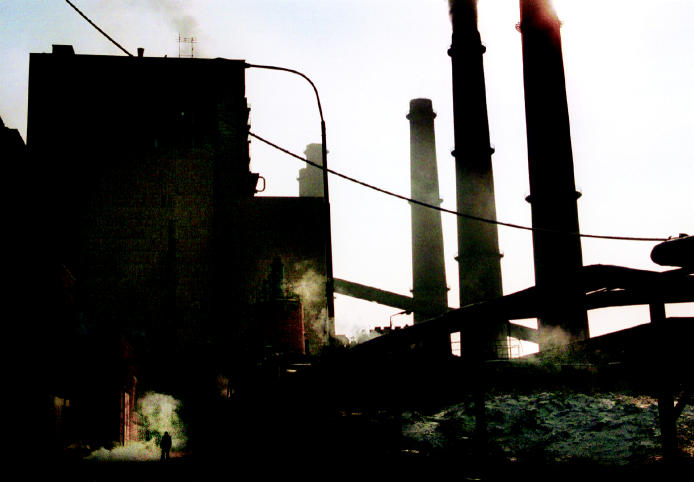
**Breaking the machinery of life.** Major industrial sites frequently become prime targets for enemy forces due to the widespread impact of their demolition. One such target was the power station in Obilic, Kosovo, which now routinely fails, cutting off power to much of the country.

**Figure:**
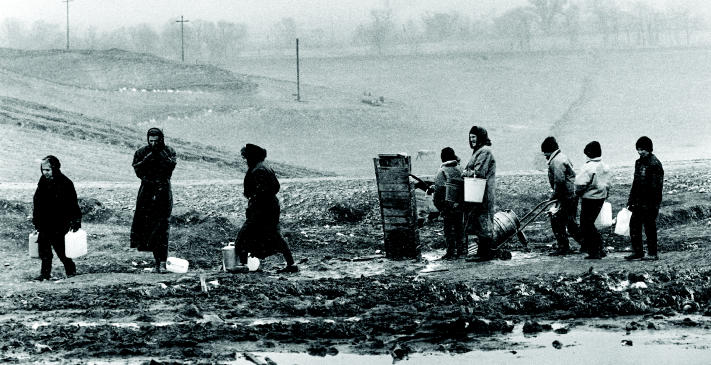
**The barest of necessities.** Chechen refugees in the Republic of Ingushetia collect water from a damaged well in Sputnik camp. There, some 8,000 people are living in 800 tents and an abandoned train.

**Figure:**
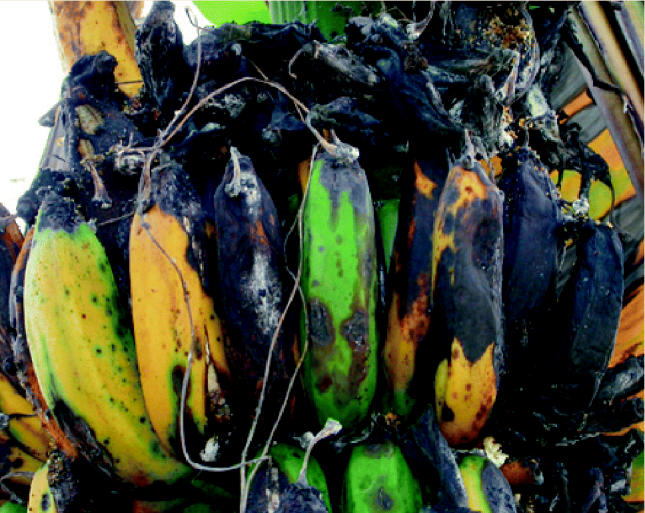
**Crop casualties.** Many Colombian farmers believe their crops, like these bananas, are being ruined by drift from herbicide spraying of illegal poppy and coca crops. Many of the ruined crops were planted at the urging of the government as alternatives to the illegal plants.

**Figure:**
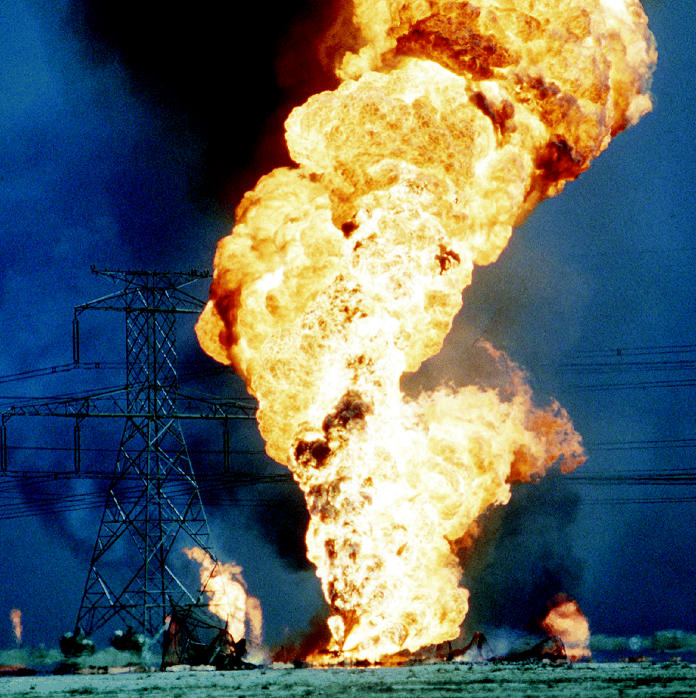
**Sending the environment up in smoke?** Oil fires set during the first Gulf War are alleged to have caused respiratory effects in both soldiers and civilians.

**Table:** Selected Modern-Day Conflicts

**Country**	**Date Begun**	**Description**	**Refugees/IDPs by the End of 2003**
**Afghanistan**	1978	Fighting among Communist government, mujahideen, Soviet Union, Taliban, and United States	2,136,000 refugees
**Angola**	1975	Marxist government versus ethnic rebels; intervention by Cuba	323,600 refugees
**Burundi**	1991	Tutsi-led government versus Hutus	531,600 refugees; 800,000 IDPs
**Cambodia**	1979	South Vietnamese versus Khmer Rouge	29,663 refugees
**Colombia**	1984	Government versus Marxist guerrillas, other insurgents, and narcotics cartels	32,793 refugees; 294,999 new IDPs in 2003
**Democratic Republic of the Congo**	1997	Government versus remnants of Hutu militias from Rwanda; involvement of Uganda, Namibia, Zimbabwe, and Angola	453,400 refugees
**Eritrea/Ethiopia**	1998	Conflict over border territory	162,196 refugees
**Indonesia**	1989	Government versus Aceh province separatists	7,491 refugees
**Iraq**	1991, 2003	U.S. invasions	368,400 refugees
**Israel**	1948	Religious/ethnic/territorial conflict	800,000 refugees
**Kosovo**	1998	Serbian government versus ethnic separatist Albanians	257,000 IDPs
**Liberia**	1990	Government versus rebel groups, then fighting among rebel groups	353,300 refugees; 227,000 new IDPs in 2003
**Myanmar**	1983	Government versus Karens and other ethnic minorities demanding autonomy	138,108 refugees
**Russia**	1994	Government versus Chechen separatists	800,000 refugees
**Somalia**	1982	Government versus rebel movement and clan guerrillas	402,200 refugees
**Sri Lanka**	1983	Government versus Tamil Tiger separatists	103,368 refugees
**Sudan**	1983	Government versus rebels; Muslims versus Christians; Janjaweed militias versus black Muslims	606,200 refugees; 4,500,000 IDPs
**Uganda**	1996	Government versus Lord’s Resistance Army	220,000 refugees

**Note:** Some conflicts have ceased, some are sporadic, and some are ongoing. Not all types of information are available for all conflicts. See sources for details. All numbers are approximate.

**Sources:** Conflict Map. Stockholm, Sweden: Nobelprize.org, The Official Web Site of the Nobel Foundation. Available: http://nobelprize.org/peace/educational/conflictmap/about.html [accessed 10 November 2004]. IRC. 2003. Mortality in the Democratic Republic of Congo: Results from a Nationwide Survey. New York, NY: International Rescue Committee. Marshall MG, Gurr TR. 2003. Peace and Conflict 2003: A Global Survey of Armed Conflicts, Self-Determination Movements, and Democracy. College Park, MD: University of Maryland, Integrated Network for Societal Conflict Research. UNHCR. 2004. 2003 Global Refugee Trends. Geneva, Switzerland: United Nations High Commissioner for Refugees.

